# Identification of Novel miRNAs, Targeting Genes, Signaling Pathway, and the Small Molecule for Overcoming Oxaliplatin Resistance of Metastatic Colorectal Cancer

**DOI:** 10.1155/2022/3825760

**Published:** 2022-09-19

**Authors:** Md Misbah, Manoj Kumar, Kuen-Huar Lee, Shing-Chuan Shen

**Affiliations:** ^1^International Ph.D. Program in Medicine, College of Medicine, Taipei Medical University, Taipei 110, Taiwan; ^2^Centre for Translational and Clinical Research, School of Chemical and Life Sciences, Jamia Hamdard, New Delhi 110062, India; ^3^Graduate Institute of Cancer Biology and Drug Discovery, College of Medical Science and Technology, Taipei Medical University, Taipei 11031, Taiwan; ^4^PhD Program for Cancer Molecular Biology and Drug Discovery, College of Medical Science and Technology, Taipei Medical University, Taipei 11031, Taiwan; ^5^Cancer Center, Wan Fang Hospital, Taipei Medical University, Taipei 11031, Taiwan; ^6^Graduate Institute of Medical Sciences, College of Medicine, Taipei Medical University, Taipei 110, Taiwan; ^7^Department of Dermatology, School of Medicine, College of Medicine, Taipei Medical University, Taipei 110, Taiwan

## Abstract

One of the globally common cancers is colorectal cancer (CRC). At present, a surgical approach remains a good option for CRC patients; however, 20% of surgically treated CRC patients experience metastasis. Currently, even the first-line used drug, oxaliplatin, remains inadequate for treating metastatic CRC, and its side effect of neurotoxicity is a major problem when treating CRC. The Gene Omnibus GSE42387 database contains gene expression profiles of parental and oxaliplatin-resistant LoVo cell lines. Differentially expressed genes (DEGs) between parental and oxaliplatin-resistance LoVo cells, protein-protein interactions (PPIs), and a pathway analysis were determined to identify overall biological changes by an online DAVID bioinformatics analysis. The ability of DEGs to predict overall survival (OS) and disease-free survival (DFS) was validated by the SPSS 22.0, using liver metastasis CRC patient samples of GSE41258. The bioinformatics web tools of the GEPIA, the Human Protein Atlas, WebGestalt, and TIMER platforms were used. In total, 218 DEGs were identified, among which 105 were downregulated and 113 were upregulated. After mapping the PPI networks and pathways, 60 DEGs were identified as hub genes (with high degrees). Six genes (*TGFB1*, *CD36*, *THBS1*, *FABP1*, *PCK1*, and *IRS1*) were involved with malaria, PPAR signaling, and the adipocytokine signaling pathway. High expressions of *CD36* and *PCK1* were associated with the poor survival of CRC patients in the GSE41258 database. We predicted specific micro (mi)RNAs that targeted the 3′ untranslated region (UTR) of *PCK1* by using miRWalk. It was found that three miRNAs, viz., miR-7-5p, miR-20a-3p, and miR-636, may be upstream targets of those genes. High expression levels of miR-7-5p, miR-20a-3p, and miR-636 were associated with poor OS of CRC patients, and the small-molecule compound, mersalyl, is a promising drug for treating oxaliplatin-resistant CRC. In conclusion, miR-7-5p miR-20a-3p, and miR-636 targeted the PCK1 biomarker in the PPAR signaling pathway, which is involved in oxaliplatin-resistant CRC. Meanwhile, mersalyl was identified as a potential drug for overcoming oxaliplatin resistance in CRC. Our findings may provide novel directions and strategies for CRC therapies.

## 1. Introduction

Colorectal cancer (CRC) is the third most common epithelial malignancy worldwide. According to the GLOBOCAN data source in 2020 in the United States, approximately 147,950 individuals were diagnosed with CRC and 53,200 patients died of this disease [1]. Surgery is the primary intervention measure for CRC patients at the primary stage of diagnosis; however, it is futile in patients who have developed distant metastasis. After surgery, approximately 20% of patients present metastatic (m)CRC micrometastasis [2, 3]. First-line treatments for mCRC patients include FOLFOX (5-FU, leucovorin, oxaliplatin, and irinotecan) and CAPEOX (oxaliplatin and capecitabine), but the response rate of mCRC patients for first-line chemotherapies is <50% [4]. Mitogen-activated protein kinase 1 (MAPK1), phosphatidylinositol 3-kinase (PI3K), and other biomarkers are currently used for CRC treatment [5, 6]. Increasing numbers of biomarkers are currently being used to diagnose and choose treatments for patients with CRC [7]. Therefore, finding useful biomarkers to identify patients who are sensitive to chemotherapy is urgently needed in the clinic.

Oxaliplatin (OXA) is a platinum-derived antitumor drug that acts to inhibit DNA replication and proliferation and induces apoptosis [8]. Nowadays, OXA resistance is a major problem, and poor survival rates are still common outcomes in mCRC patients. Several previous studies showed that many pathways and molecules are involved in OXA resistance in mCRC including malaria, peroxisome proliferator-activated receptor (PPAR) signaling, and the adipocytokine signaling pathway. The PPAR signaling pathway is involved in colorectal carcinoma cell death and the development of CRC. PPAR*γ* and PPAR*δ* which are involved in regulated programmed cell death are mediated by caspase-3 and survivin [9, 10].

Many biomarkers are involved in this pathway including phosphoenolpyruvate carboxykinase 1 (PCK1), fatty acid binding protein 1 (FABP1), and CD36. Mersalyl is an organomercurial compound that induces vascular endothelial growth factor (*VEGF*) gene expression and activates collagenase under invasive conditions where plasmin formation or activity is inhibited [11, 12]. Some herbal supplements, such as resveratrol, quercetin, and thymoquinone, possibly enhance sensitivity to OXA therapy. There is an urgent requirement to improve therapeutic regimens for CRC. Seeking reliable biomarkers is a way to foresee possible consequences related to OXA treatment and considerations for therapeutic management.

The Gene Expression Omnibus (GEO) database is a very important dataset for identifying novel biomarkers and novel drug therapies for cancers and other diseases [13, 14]. In addition, protein-protein interactions (PPIs) and Kyoto Encyclopedia Genes and Genomes (KEGG) analyses help identify pathways involved in chemotherapeutic resistance in CRC patients [15, 16]. GEPIA, the Human Protein Atlas (HPA) database, The Cancer Genome Atlas (TCGA) database, and TIMER were also used to determine gene expression levels of differentially expressed genes (DEGs) [17].

PCK1 is a gluconeogenic enzyme involved in gluconeogenesis and lipogenesis processes in hepatocellular carcinoma (HCC). The *PCK1* gene was also found in many cancerous organs, including the colon, skin, and lungs, and is also involved in anabolic metabolism and cell proliferation [18, 19]. In an earlier study, PCK1 mediated sterol regulatory element-binding protein 1(SREBP1) activation in esophageal cancer (ESCC) and non-small-cell lung cancer (NSCLC) [20]. The gluconeogenic gene, *PCK1*, is located in the endoplasmic reticulum (ER), where PCK1 acts like a protein kinase enzyme for the use of guanosine 5′-triphosphate (GTP), rather than adenosine triphosphate (ATP) as a phosphate donor to phosphorylate INSIG1/2. This phosphorylation process reduces the binding of oxysterol to INSIG1/2, thus activating SREBP-mediated lipogenesis for tumor growth [21, 22].

Micro (mi)RNAs are small noncoding RNAs that regulate gene expressions by binding to the 3′ untranslated region (UTR) of their target messenger (m)RNAs for translational repression and/or degradation and can also influence oncogene factors and mechanisms, resulting in stimulation of OXA chemoresistance. Oncosuppressive miRNAs can enhance the sensitivity of cancer cells to OXA chemotherapy and activate apoptosis and cell cycle arrest and induce cell viability and tumor progression [23]. miRNAs are very commonly used in chemotherapy-resistance studies to develop sensitivity [24].

This study is aimed at identifying biomarkers of drug response and investigating mechanisms of drug resistance to the two chemotherapeutic drugs, OXA, and irinotecan (the active metabolite of which is SN-38), in CRC using both drug-resistant and drug-sensitive parenteral colon cancer cell lines. Overall, the study was designed to identify a microarray analysis to predict OXA-resistant genes and underlying pathways in CRC.

## 2. Methods

### 2.1. Data Resources

We used the GEO database (available at http://www.ncbi.nlm.nih.gov/geo/) to identify DEGs. The gene expression profile of GSE42387 was downloaded from GEO which was sequenced on the GPL16297 platform of the Agilent-014850 Human Genome CGH Microarray 4x44K G4112F (Agilent Technologies, Santa Clara, CA, USA). Three colon cancer cell lines of HCT116, HT29, and LoVo present in the GSE42387 database were exposed to an increasing concentration of OXA or SN-38 for 9 months in vitro to generate subcell lines with acquired resistance. Gene expressions of the parental and resistant cell lines grown in drug-free media were compared to detect any differences linked to chemotherapeutic resistance.

### 2.2. Data Preprocessing and DEGs

The GSE42387 dataset contained three control LoVo (metastatic) CRC cell lines and three OXA-resistant LoVo (metastatic) CRC cell lines. For the differential expression analysis, we used the GEO2R-friendly tool and recalculated the data [25]. The GEO2R online tool applies R language for the GEO querry and limma packages which were used to examine gene expressions. The LoVo parental cell lines vs. OXA-resistant cell lines were selected to identify DEGs between resistant and sensitive cell lines. The *t*-test method was applied to calculate *p* values of these genes. Then, Benjamin and Hochberg's method was performed to calculate adjusted *p* values (of the false discovery rate, FDR) of the DEGs with a log fold change (FC) of >1 or <-1 and an FDR of <0.05 selected [26].

### 2.3. Hierarchical Clustering Analysis

Furthermore, after obtaining expression values from the gene expression profile, a bidirectional hierarchical clustering heat map was constructed with the CIMminer web tool [27].

### 2.4. Construction of a Protein-Protein Interaction (PPI) Network

A PPI network was constructed using the online web tool STRING (http://www.string-db.org/) [28]. This web tool provides known protein and predicted protein interactions which are derived from four sources including genomic context, coexpression, high-throughput experiments, and previous knowledge. A score of 0.4 (medium confidence) was selected as the cutoff criterion. PPI pairs were input into Cytoscape software (ver. 3.4.0, http://www.cytoscape.org) and analyzed with the CytoNCA app in Cytoscape. Hub genes (highly connected genes) were identified by calculating the degree value (number of lines connecting the genes) with a cutoff of two or more.

### 2.5. Pathway and Enrichment Analysis

The Database for Annotations, Visualization, and Integrated Discovery (DAVID bioinformatics, https://david.ncifcrf.gov/) was used to differentiate gene expressions by their cellular components, molecular functions, and biological processes using the resource from Gene Ontology (GO, available at http://www.geneontology.org/) [29]. An enrichment analysis was conducted using DAVID and pathways referenced from the Kyoto Encyclopedia of Genes and Genomes (KEGG, http://www.genome.jp/kegg/) database website with an FDR of <0.25 as a cutoff point [30]. A Gene Set Enrichment Analysis (GSEA) was used for confirmation of KEGG pathways as well [31].

### 2.6. Expression Analysis of DEGs in CRC

GEPIA databases available were used to check expression levels of DEGs in normal and CRC tissues. The threshold absolute log base 2 of the fold change (Log2FC) was set to 1 and was analyzed using the *q* value set to 0.05 [32].

### 2.7. Protein Expression Analysis of DEGs in CRC

The intensity of DEGs protein expressions in CRC tissues in the human body was investigated using the Human Protein Atlas (HPA) database (https://www.proteinatlas.org/). More than 700 antibodies to human proteins available are matched with 400,000 high-resolution pictures which are available in the HPA database [33, 34]. The following criteria were used to evaluate each intensity and fraction combination which was automatically converted into a protein expression level score: negative—not detected; weak—not detected; weak combined with either 25%~75% or 75%—low; moderate—low; moderate combined with either 25%~75% or 75%—medium; strong—medium, and strong combined with either 25%~75% or 75%—high.

### 2.8. Validation of OXA-Resistant Genes

To examine candidate genes' roles, we conducted a survival analysis. A Kaplan-Meier (KM) survival analysis was constructed using the GSE41258 cohort of the GEO database [35]. We selected 243 CRC patients for overall survival analysis and 189 CRC patients for disease-free survival with mutation and RNA sequence data divided into high expression and low expression by median value with the use of log rank *p* value. Moreover, we also examined cox regression analysis. Furthermore, we also predicted survival as related to miRNAs using the GSE29623 cohort and SPSS ver. 22.0 (SPSS, Chicago, IL, USA, http://www-01.ibm.com).

### 2.9. Prediction of miRNAs for DEGs

We also predicted miRNAs using the GSE29623 cohort study, 143 miRNA samples in metastatic colon adenocarcinomas vs. the miRWalk website's 1216 miRNA PCK1 3′ UTR. We chose a target score more than 0.70 for the PCK1 gene in miRWalk web tools. Furthermore, a Venn diagram was used to predict common miRNAs and their gene targets [36].

### 2.10. Clinical Examination of miRNAs

For clinical examination of differentially expressed miRNAs, a survival analysis was performed using CRC patients. SPSS ver. 22.0 was used to plot KM curves (SPSS, Chicago, IL, USA, http://www-01.ibm.com), boxplots, and a receiver operating characteristic (ROC) curve. Tissue expressions of miRNAs including GSE29623 with a total of 65 cases and GSE126093 with 20 cases in the CRC Metabase were chosen. Expression profiles were compared based on low or high values with the Mann–Whitney test and *p* value (with *p* < 0.05 considered significant).

### 2.11. Small-Molecule Drug-Targeting Therapy for DEGs

The web-based GEne SeT AnaLysis Toolkit (WebGestalt, http://www.webgestalt.org), an integrated system for gene analysis, was used to predict the drugs associated with DEGs [37].

### 2.12. Immune Infiltration Analysis of DEGs in CRC

For the immune infiltration analysis, we used the TIMER database (http://timer.cistrome.org/), a comprehensive database that provides analysis of immune infiltrates in various types of cancer [38, 39]. We utilized this database to uncover the involvement of DEGs in immune infiltrates in CRC. Relationships of gene expressions and estimated infiltrate values were presented using scatterplots, and the level of significance of the correlation was *p* < 0.05.

## 3. Results

### 3.1. DEGs in GSE42387

More than 100 DEGs, hub genes, and many pathway-related genes were identified as being associated with CRC [40]. Data extracted from the GPL16297 microarray platform using the GEO2R tool consisted of 32,706 probe sets. In total, 218 DEGs were predicted to be related to OXA resistance after calculating logFC and FDR values, and among these, 105 DEGs were downregulated and 113 DEGs were upregulated (Supplementary Tables 1 and 2).

**Figure 1:**
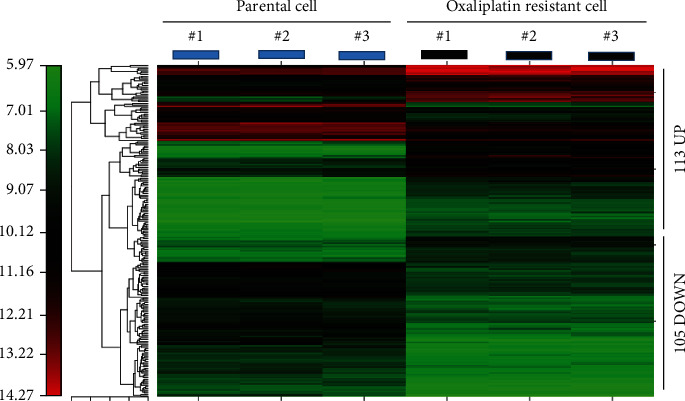
Heat map showing upregulated and downregulated genes in oxaliplatin-resistant colon cancer tumors. A bidirectional hierarchical clustering heat map was constructed using the CIMminer web tool. Expression values are log fold changes (>1.0 or <-1.0, with a false detection rate of <0.05) between corresponding oxaliplatin-resistant LoVo cells and parental LoVo cells. Red represents upregulation, black represents no change in expression level, and green represents downregulation.

The CIMminer online web tool was used to design a heat map to predict the bidirectional hierarchical clustering of downregulated and upregulated DEGs, and it is presented in Figure 1.

### 3.2. Protein-Protein Interaction (PPI) Network

**Figure 2:**
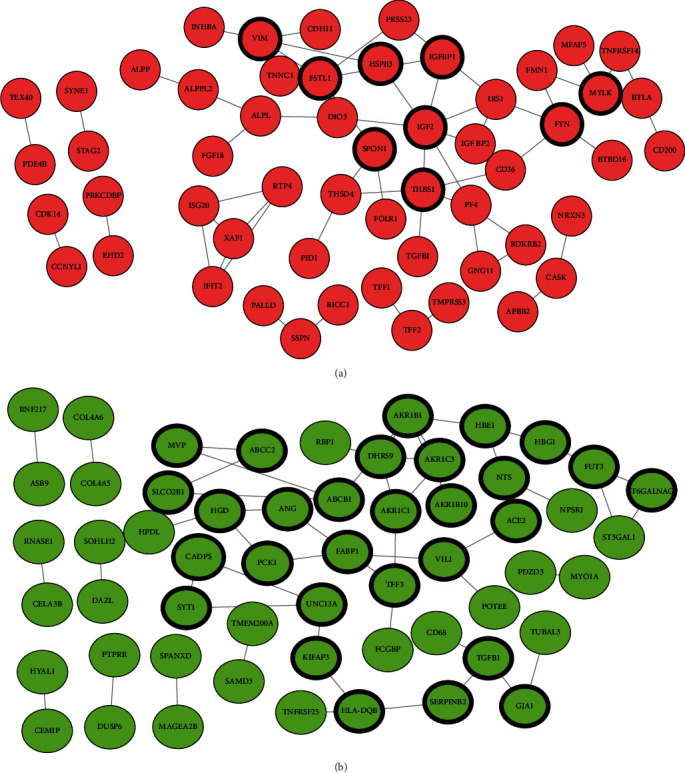
Protein-protein interaction (PPI) network of differentially expressed genes (DEGs). (a) Upregulated genes and (b) downregulated genes. PPI pairs were imported into Cytoscape software as described in Methods. Red nodes represent upregulated genes while green nodes represent downregulated genes. The lines represent the interactive relationship between nodes. The highlighted DEGs represent a degree of ≥2.

The DEGs were loaded into the STRING database (https://string-db.org/) to obtain PPI pairs and then imported into Cytoscape software to identify hub genes. As shown in Figure 2(a) and Table 1, the upregulated DEG network contained 30 DEGs which were identified as hub genes. The downregulated network contained 30 DEGs which were identified as hub genes (Figure 2(b), Table 2).

### 3.3. KEGG Pathway Analysis

**Figure 3:**
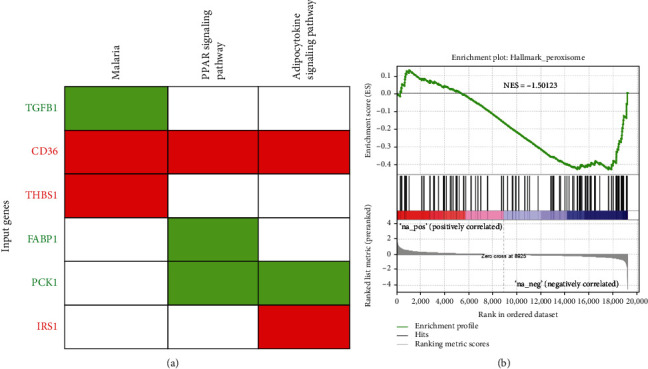
Significant KEGG pathways and genes involved. (a) Gene enrichment analysis showing that KEGG pathways were significantly enriched in oxaliplatin-resistant LoVo xenograft tumors and genes involved in the pathways (the pathways are in the order of their enrichment from left to right), *p* < 0.05). **(**b**)** Gene Set Enrichment Analysis (GSEA) showing the peroxisome Hallmark enrichment score.

The 60 hub genes were applied to obtain KEGG pathways using the DAVID online tool. Six KEGG pathways (malaria, peroxisome proliferator-activated receptor (PPAR) signaling, adipocytokine signaling pathway, metabolic pathways, folate biosynthesis, and insulin resistance) were identified. Among them, malaria, PPAR signaling, and the adipocytokine signaling pathway reached statistical significance (FDR value of <1 and *p* < 0.05) and included *TGFB1*, *CD36*, *THBS1*, *FABP1*, *PCK1*, and *IRS1* (Table 3). Expression patterns of candidates for malaria, PPAR signaling, and adipocytokine signaling pathway were plotted as a heat map using CIMminer (Figure 3). The three genes, *CD36*, *THBS1*, and *IRS1*, were upregulated, and the other three genes, *TGFB1*, *FABP1*, and *PCK1*, were downregulated. Furthermore, using GSEA, peroxisome pathways were preferable as a significant biological process related to OXA-resistant CRC (Figure 3(b), normalized enriched score (NES) = −1.50123).

### 3.4. Expression Levels of FABP1, CD36, IRS1, PCKI, THBS1, and TGFB1 in *CRC*

**Figure 4:**
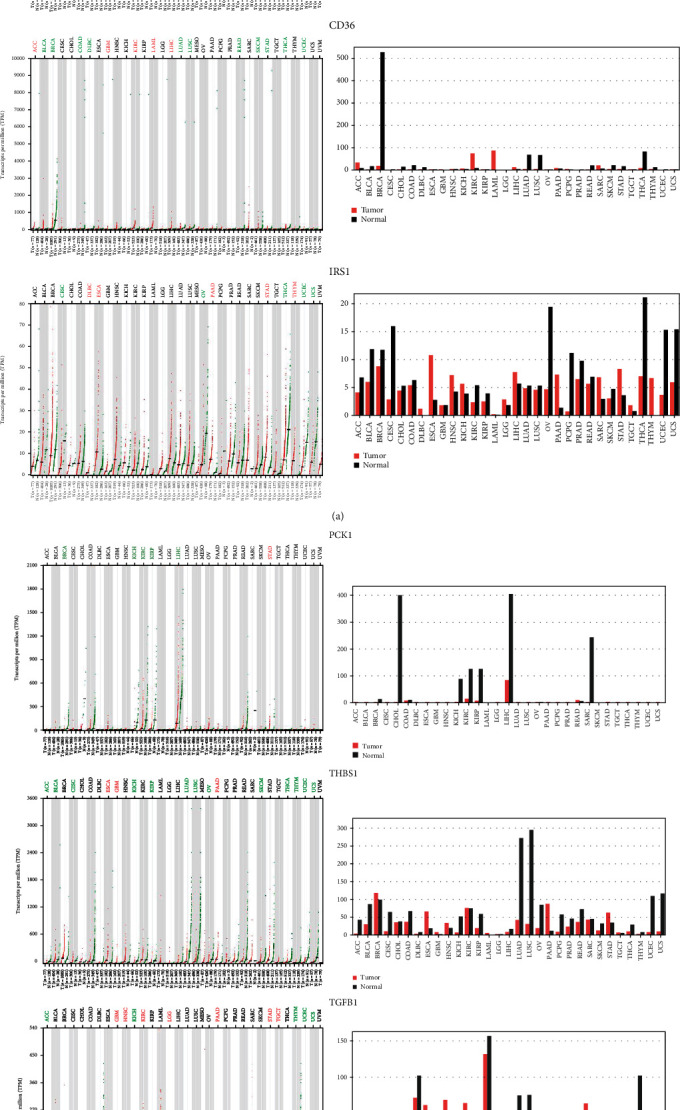
Gene expression profiles of differentially expressed genes (DEGs) of (a) *FABP*, *CD36*, and *IRS1* and (b) *PCK1*, *THBS1*, and *TGFB1*. These were evaluated in 27 TCGA tumor samples vs. normal tissues using the GEPIA web tool. The black bar indicates normal tissues, while the red bar indicates expressions of DEGs in tumor tissues. Each GTEx normal data point (green) and its matched TCGA tumor (red) used TPM (transcripts per million (log2 (TPM + 1)). *X* axis: number of tumor and normal samples. ACC: adrenocortical carcinoma; BLCA: bladder urothelial carcinoma; BRCA: breast invasive carcinoma; CESC: cervical squamous cell carcinoma and endocervical adenocarcinoma; CHOL: cholangiocarcinoma; COAD: colon adenocarcinoma; DLBC: lymphoid neoplasm diffuse large B-cell lymphoma; ESCA: esophageal carcinoma; GBM: glioblastoma multiforme; HNSC: head and neck squamous cell carcinoma; KICH: kidney chromophobe; KIRC: kidney renal clear cell carcinoma; KIRP: kidney renal papillary cell carcinoma; LAML: acute myeloid leukemia; LGG: brain lower-grade glioma; LIHC: liver hepatocellular carcinoma; LUAD: lung adenocarcinoma; LUSC: lung squamous cell carcinoma; MESO: mesothelioma; OV: ovarian serous cystadenocarcinoma; PAAD: pancreatic adenocarcinoma; PCPG: pheochromocytoma and paraganglioma; PRAD: prostate adenocarcinoma; READ: rectum adenocarcinoma; SARC: sarcoma; SKCM: skin cutaneous melanoma; STAD: stomach adenocarcinoma; TGCT: testicular germ cell tumors; THCA: thyroid carcinoma; THYM: thymoma; UCEC: uterine corpus endometrial carcinoma; UCS: uterine carcinosarcoma.

**Figure 5:**
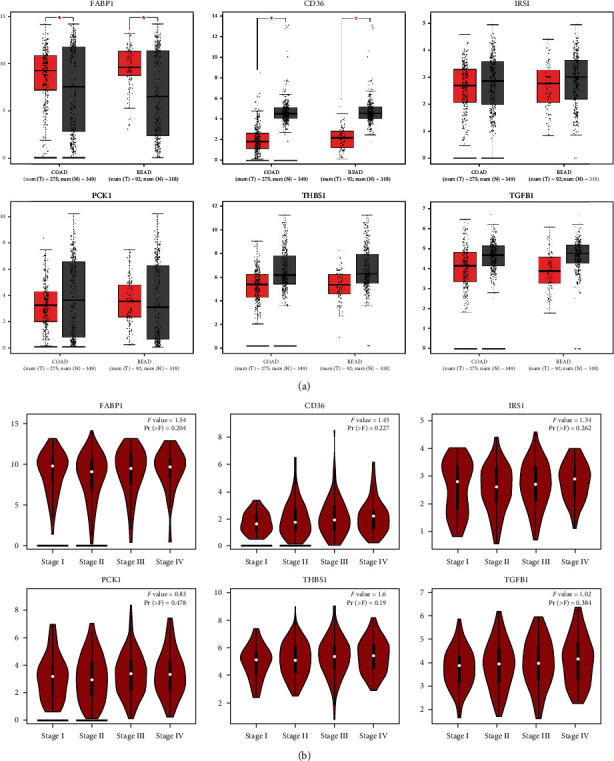
Gene expression profiles of (a) *FABP*, *CD36*, *IRS1*, *PCK1*, *THBS1*, and *TGFB1* in colorectal cancer (CRC). Boxplot showing transcriptional levels of differentially expressed genes (DEGs) in colon adenocarcinoma (COAD) (*n* = 275) vs. normal samples (*n* = 349) and rectal adenocarcinoma (READ) (*n* = 92) vs. normal tissues (*n* = 318) using the GEPIA web tool based on TCGA database. Black colors show transcriptional levels in normal tissues, while red colors show DEG transcriptional levels in COAD and READ tissues. One-way ANOVA was used for the differential analysis with a statistically significant value of *p* < 0.05. (b) All stages of CRC are shown for cancer progression of the five DEGs. A violin plot shows different stages of cancer with log2 (transcripts per million (TPM) + 1) of genes in stages I to IV. A *t*-test was used with the statistically significant *p* < 0.05. The *Pr*(>*F*) < 0.05, followed by Student's *t*-test.

Moreover, we also examined expression levels of *FABP1*, *CD36*, *IRS1*, *PCKI*, *THBS1* and *TGFB1* in CRC with a GEPIA web tool analysis. According to the GEPIA analysis (Figures 4 and 5), *FABP1* and *CD36* had significant and different expressions in CRC as shown in a boxplot of normal vs. colorectal adenocarcinoma, results showed significant differences of expression in CRC (Figures 4(a) and 5(a)), while *IRS1*, *PCK1*, *THBS1*, and *TGFB1* showed no significant differences of expression in CRC.

Moreover, we also examined associated expression levels of these six genes in clinicopathological patients. According to Figure 5(b), we found that mRNA expression levels of *PCK1* significantly differed in different tumor stages of CRC. However, expression levels of *FABP1*, *CD36*, *IRS1*, *THBS1*, and *TGFB1* did not significantly differ in various tumor stages. The *PCK1* result is consistent with a previous study that showed that it was involved in carcinogenesis but was not involved in the stages of CRC [41].

### 3.5. Protein Expression Levels of FABP1, CD36, IRS1, PCKI, THBS1, and TGFB1 in CRC

**Figure 6:**
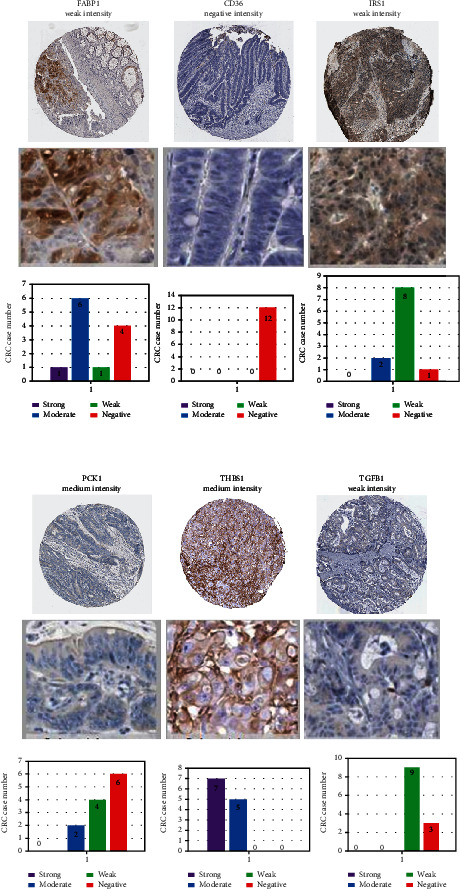
Protein expressions of six genes. The protein expression analysis used the HPA database of colorectal cancer (CRC) tissue samples. IHC images show the intensity and staining of differentially expressed genes (DEGs). Manual scoring of IHC data for staining intensity (negative, weak, moderate, or strong) and proportion of stained cells (>75%, 25%~75%, or 25%) as determined by the protein expression score.

Additionally, protein expression levels of these six biomarkers in 12 tissue samples of CRC patients were also validated by the HPA database of IHC images of immunoreactivity expression in cancer specimens (Figure 6). This figure shows manual scoring of IHC data of the staining intensity (negative, weak, moderate, or strong), and the proportion of stained cells (>75%, 25%~75%, of <25%) was used to determine the protein expression score. PCK1 and THBS1 protein expression levels exhibited moderate staining in CRC tissues and CD36 showed negative intensity. Overall FABP1, TGFB1, and IRS1 showed weak intensities. However, PCK1 showed moderate intensity with a >75% proportion, while THBS1 showed moderate intensity with 25%~75% proportion. That is why we considered PCK1 to be a more promising biomarker of CRC development.

### 3.6. Genes Associated with CRC Patient Survival

**Figure 7:**
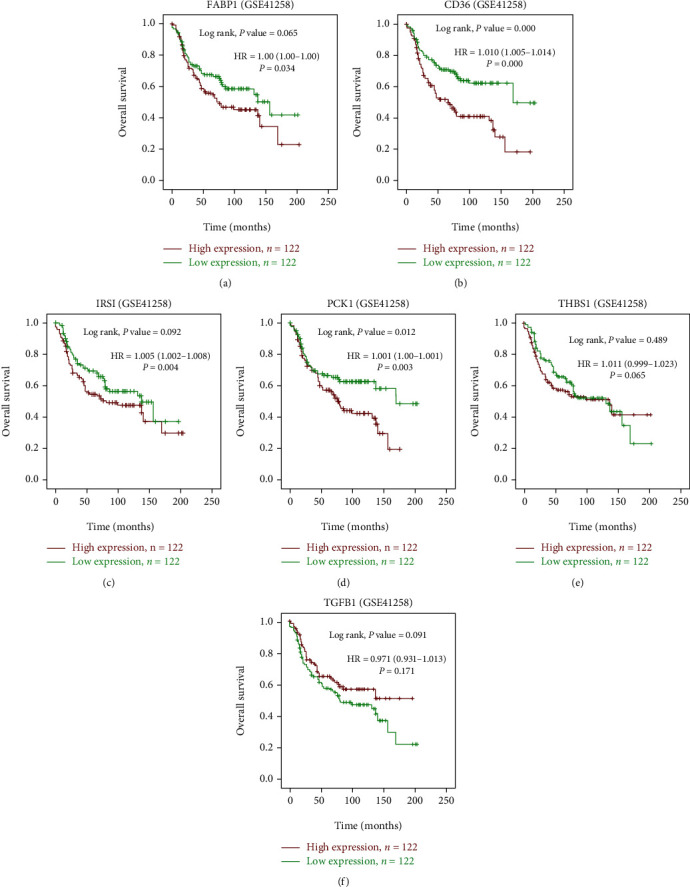
Kaplan-Meier survival curves presenting the prognostic relationship between high and low expressions of specific genes to overall survival (OS) using the GSE41258 database patient samples. (a) *FABP1* (b) *CD36*, (c) *IRS1* (d) *PCK1*, (e) *THBS1*, and (f) *TGFB1* expressions. Survival curves were plotted using SPSS 22.0. Specific differentially expressed gene (DEGS) expression levels were selected by the median value. Results are visually presented by Kaplan-Meier survival plots, and *p* values were calculated using log-rank statistics. Number of Patient (*n*) = 244, *p* = log-rank *p* value with *p* < 0.05 considered significant

**Figure 8:**
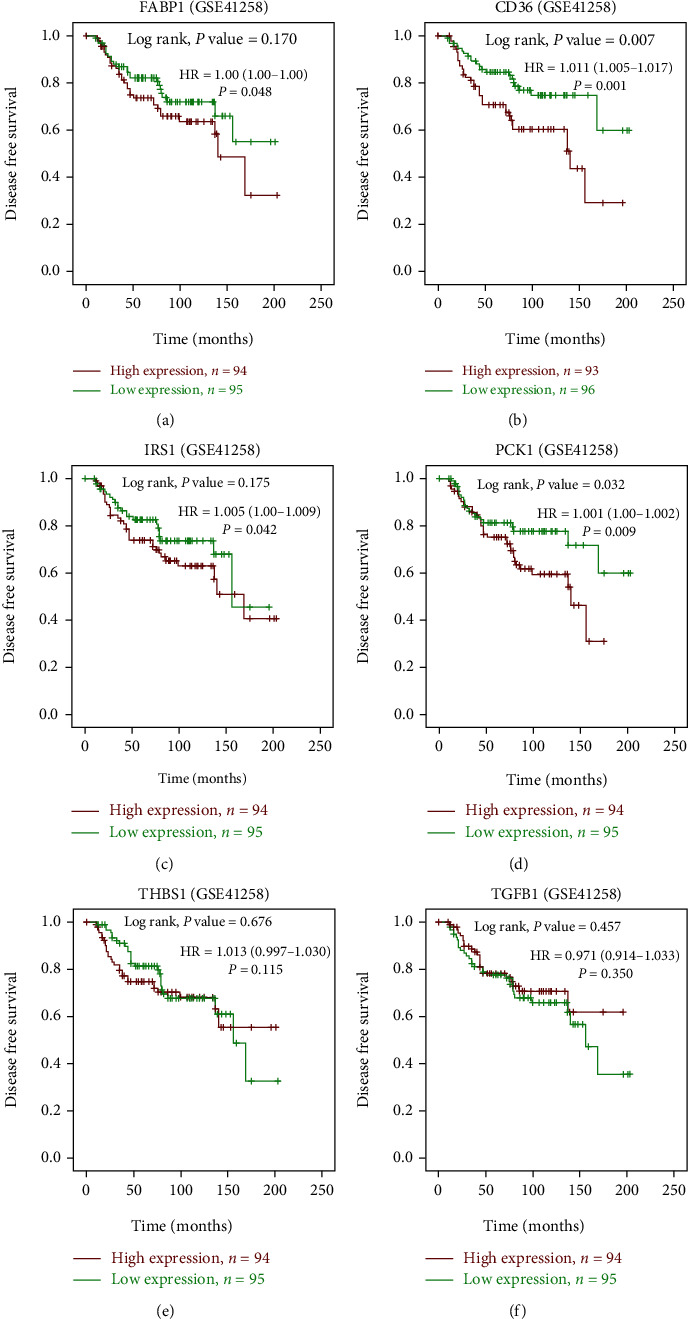
Kaplan-Meier survival curves presenting prognostic relationships between high and low expressions of specific genes to disease-free survival (DFS) using the GSE41258 database patient samples. (a) *FABP1* (b) *CD36*, (c) *IRS1* (d) *PCK1*, (e) *THBS1*, and (f) *TGFB1*expressions. Survival curves were plotted using SPSS 22.0. Specific differentially expressed gene (DEG) expression levels were selected by the median value. Results are visually presented by Kaplan-Meier survival plots, and *p* values were calculated using log-rank statistics. Patient number(*n*) = 189;*p* = log‐rank *p*value, with*p* < 0.05considered significant.

Furthermore, we conducted a survival analysis for candidate targets using a cohort of GSE41258 liver metastasis CRC patient samples with the use of SPSS 22.0 to draw a Kaplan-Meir Plot. As shown in Figures 7 and 8, high levels of *CD36* and *PCK1* were associated with poor OS and DFS of CRC patients, while *THBS1*, *FABP1*, *TGFB1*, and *IRS1* were not. We used the log rank test *p* value < 0.05 followed by multiple testing the cox regression analysis. We found that *PCK1* is a consistent result from survival analysis and protein expression immunohistochemistry results.

### 3.7. Identification of Potential miRNA Targets for Candidate Genes

**Figure 9:**
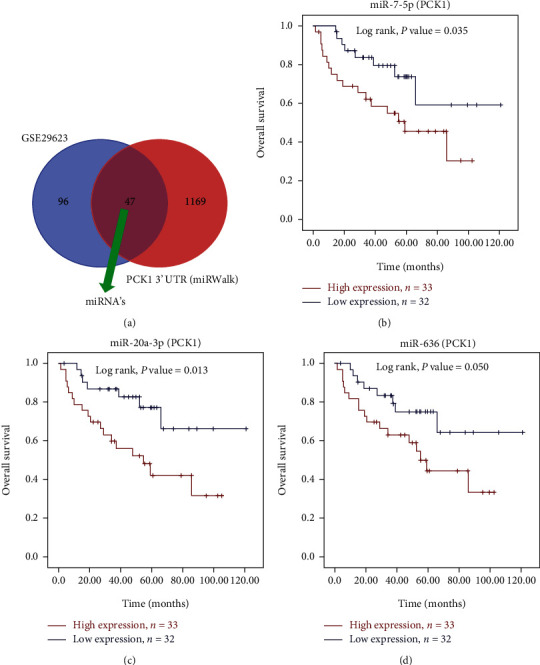
Kaplan-Meier survival curves presenting prognostic relationships between high and low expressions of specific micro (mi)RNAs to overall survival (OS) using the GSE29623 database patient samples. (a) Venn plot method for prediction of miRNAs (b) miR-7-5p, (c) miR-20a-3p, and (d) miR-636 expressions. Survival curves were plotted using SPSS 22.0. Specific miRNA expression levels were selected by the median value. Results are visually presented by Kaplan-Meier survival plots, and *p* values were calculated using log-rank statistics. Patient number(*n*) = 65;*p* = log‐rank *p*value, with*p* < 0.05considered significant.

To determine upstream regulators of those candidates of GSE29623, miRWalk web tools were applied to predict miRNAs for those candidates. We found that 47 miRNAs targeted *PCK1* (Figure 9(a) and supplementary Table 3). Finally, we focused on 8 miRNAs for further analysis (Supplementary Table 4).

### 3.8. miRNAs Associated with Survival of CRC Patients

Moreover, we also validated the OS of targeted miRNA expression levels from CRC tissue samples of the GSE29623 database using SPSS ver. 22.0 to draw a KM plot. We further verified correlations of the 8 miRNAs with clinical outcomes of CRC. As shown in Figures 9(b)–9(d), high expressions of miR-7-5p, miR-20a-3p, and miR-636 were associated with poor OS while the other 5 miRNAs were not significantly correlated with the OS of CRC patients (Supplementary Table 4).

### 3.9. Clinical Validation of miR-7-5p, miR-20a-3p, and miR-636 in CRC Patients

The GSE126093 GEO dataset comprising miRNA profiles from tissues of 20 CRC patients was used. We found that the expression levels of miR-7-5p, miR-20a-5p, and miR-636 were higher in CRC patients and the Mann–Whitney test *p* values were <0.05 (Supplementary Figures 1A-C). Furthermore, we utilized a sensitivity test (ROC analysis) (Supplementary Figures 2A-C). From the overall results, we picked three miRNAs due to their significant associations with OS. Only a high level of PCK1 was negatively regulated by high levels of miR-7-5p miR-20a-5p and miR-636 to activate the PPAR pathway.

### 3.10. Functional Interactions and Pathway Enrichment

**Figure 10:**
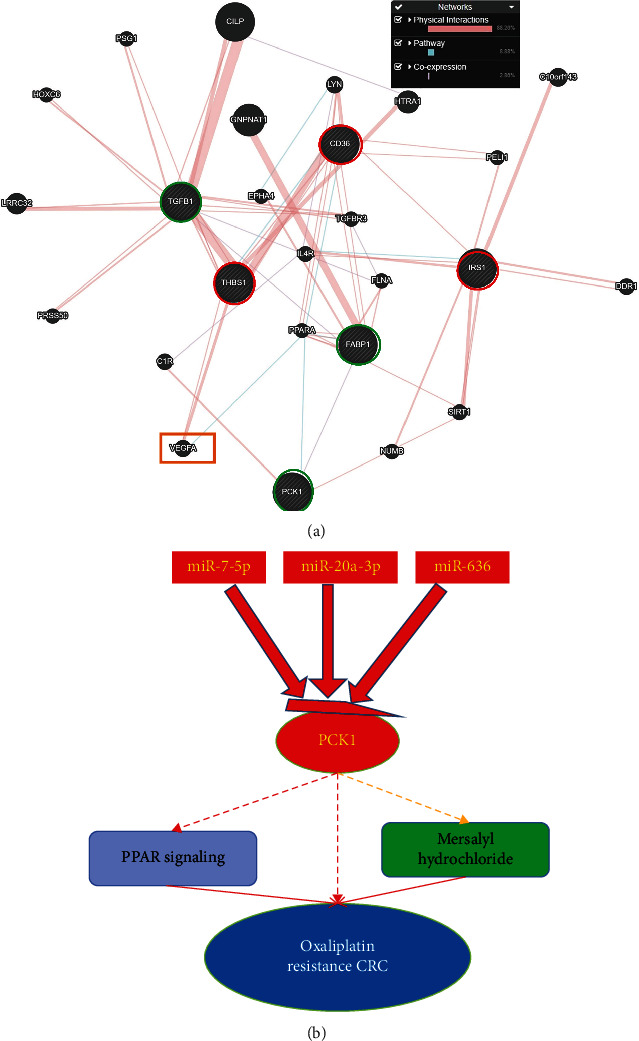
Gene interaction network (a) and pathway enrichment summary of common micro (mi)RNA targets. In (a), input genes are indicated by stripes with green circles representing downregulated genes and red representing upregulated genes in oxaliplatin-resistant colorectal cancer (CRC), while in (b), a schematic summary shows possible interactions of miRNAs and their oxaliplatin-resistant targets. The red background represents upregulation, and the green background indicates downregulation in oxaliplatin-resistant CRC, revealing significant expression in the respective validation dataset. Thick dashed black lines represent significant interactions, and thin black lines represent nonsignificant interactions. Oxaliplatin is represented by a blue background.

We used the GeneMANIA online web tool to explore functional interactions of miRNA targets with each other and DEGs of the reactome. The PPAR pathway was used to analyze the functional roles of these molecules. The interaction network included 20 other related genes in addition to the two targets (*PCK1* and *IRS1*) that were entered, and there were 144 links in total. Two interaction types were involved, and coexpression was the most frequent type of interaction (2.88%) (Figure 10(a)). High expressions of miR-7-5p, miR-20a-5p, and miR-636 targeting PCK1 suppressed OXA-resistant CRC through activation of the PPAR pathway. Overall interactions of miRNAs with target genes and their mechanisms are summarized in Figure 10(b).

### 3.11. Association of the PCK1 Gene with Immune Cell Infiltration

**Figure 11:**
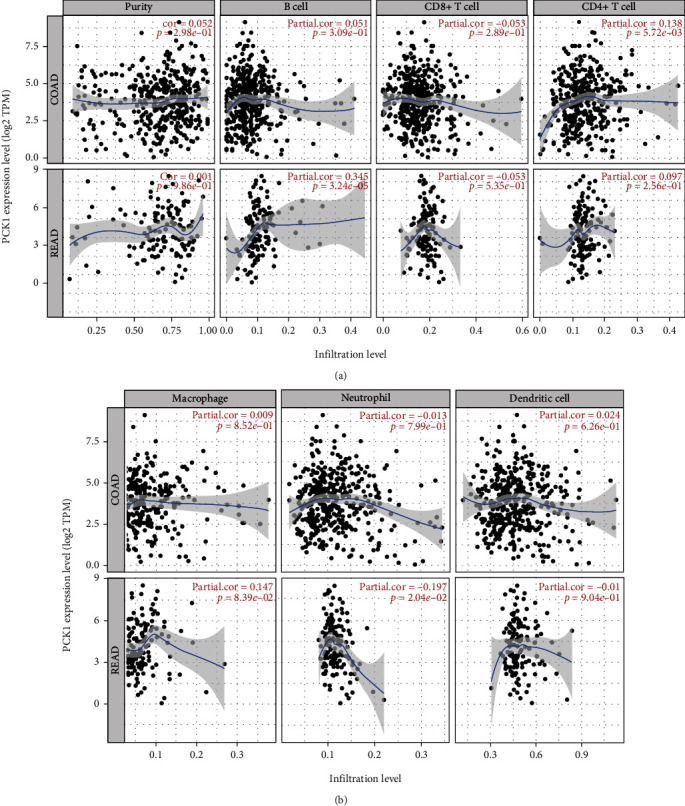
Immune filtration of the phosphoenolpyruvate carboxykinase 1 (*PCK1*) gene. Spearman's correlations between the differentially expressed *PCK1* gene and immune cell infiltration in (a) colon adenocarcinoma (COAD) and (b) rectal adenocarcinoma (READ) patients. The TIMER web tool was used for the analysis of correlations between immune infiltration of the immune cell markers of B cells, CD4^+^ cells, CD8^+^ cells, T cells, macrophages, neutrophils, and dendritic cells vs. the *PCK1* gene. Statistical significance was accepted at *p* < 0.05 for Spearman's correlations.

We also examined the relationship of the *PCK1* gene with immune cell infiltration and inflammatory responses in CRC patients. We used the TIMER database to predict whether *PCK1* gene expression was linked to immune infiltration in CRC patients (Figure 11, Supplementary Figure 3). Results showed correlations of *PCK1* with a cluster of differentiation 4-positive (CD4^+^) T cells and macrophages, while neutrophils were negatively correlated in colon adenocarcinoma (COAD) and rectal adenocarcinoma (READ) patients.

### 3.12. Drug Predictions

**Figure 12:**
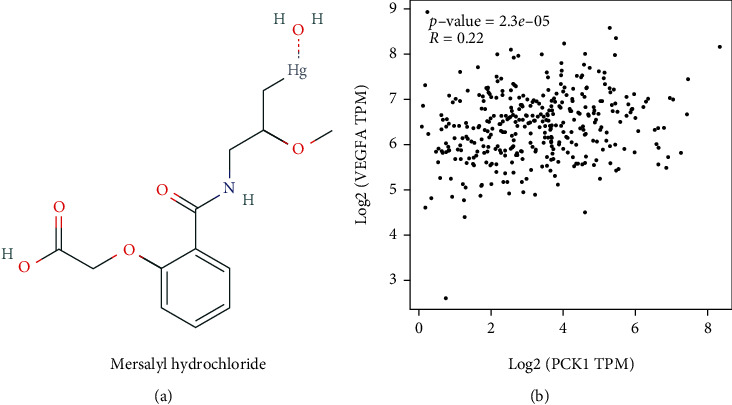
(a) Mersalyl hydrochloride chemical structure. (b) Correlation between PCK1 and VEGFA. The correlation was *R* = 0.22, and the *p* value was <0.05, which was significant.

**Figure 13:**
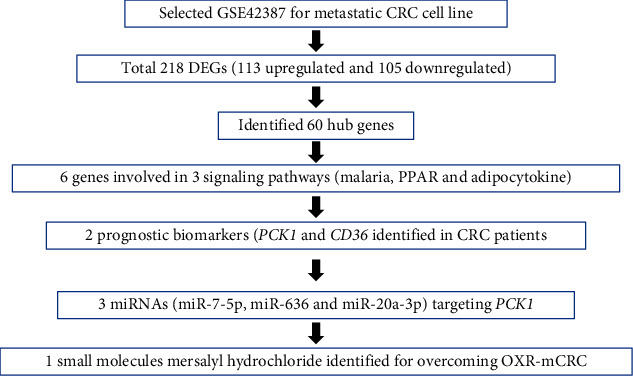
Schematic diagram.

The two genes identified by the survival analysis were used for drug predictions via the web-based GEne SeT AnaLysis Toolkit (WebGestalt, http://www.webgestalt.org). The *PCK1* gene significantly (*P* = 5.35*e* − 3) targeted mersalyl small molecules; hence, it was identified as a druggable gene that can be targeted for developing new drugs (Table 4). We also show the chemical structure of mersalyl in Figure 12(a) and the correlation between *PCK1* and VEGFA in Figure 12(b). The correlation value was *R* = 0.22 and *p* = 2.3*e* − 05. The overall study flow is shown in Figure 13.

## 4. Discussion

Our study revealed that *PCK1* is an important antitumor mRNA and inhibitor of tumor progression. *PCK1* was downregulated in OXA-resistant CRC and functionally suppressed the drug-resistant phenotype. miR-20a-3p, miR-636, and miR-7-5p overexpression resensitized the OXA response by competitively binding the PCK1 mRNA 3′ UTR, leading to PPAR signaling of OXA-resistant CRC. In this study, previously published mRNA expression datasets on OXA resistance of CRC in the GEO database were used to identify DEGs [42]. A computational analysis was performed by defining DEGs that were correlated with miRNAs. KEGG pathway enrichment analyses were identified and a PPI network was created to screen for hub genes. Furthermore, OS and DFS were evaluated to identify CRC patient survival biomarkers [43].

We explored the potential mechanisms of *PCK1* that mediated the reversal of chemoresistance by focusing on likely miRNAs. Herein, we identified that high expression levels of miR-20a-3p, miR-636, and miR-7-5p were associated with poor OS of CRC patients. Recently, miRNAs have been highly investigated for cancer treatment. But some previous studies identified negative relations between certain miRNAs and PCK1. For example, high expression of miR-33b negatively regulated PCK1 in human hepatic cells and caused a reduction in glucose production, and our study also showed consistent results in which high expression of miR-7-5p, miR-636, and miR-20a-3p negatively regulated PCK1 in CRC patients. Another example is bta-miR-26a which also reduced the abundance of PCK1 [44, 45]. Moreover, the oncosuppressor miR-20a-3p was identified in melanoma, tongue squamous cell carcinoma, and hepatocellular carcinoma, and the oncosuppressor miR-636 was also identified in lung cancer, nasopharyngeal carcinoma, cervical cancer, endometrial cancer, ovarian cancer, and hepatocellular carcinoma [46–56]. It was reported that high miR-20a-3p and miR-636 expression were correlated with poor OS of CRC patients and this was consistent with our results. In a previous study, miR-7-5p modulated OXA resistance in HCC [57]. Downregulated miR-7-5p inhibited tumor growth, migration, invasion, and proliferation and induced apoptosis in CRC [58, 59].

OXA-resistant metastatic CRC is a major problem in the clinical management of CRC and causes neurotoxic side effects due to the OXA treatment of CRC patients. This is due to the difficulties associated with early detection of the disease and the development of acquired therapeutic resistance leading to ineffective treatment in patients with metastatic disease [60–63]. Therefore, the etiological factors and mechanisms of acquired OXA resistance must be well studied to increase survival rates and prevent disease recurrence [61]. Microarray technology is commonly used to identify therapeutic targets for the diagnosis and prognosis of cancers [15, 64]. In a previous study, PCK1 was found to be a key enzyme in the gluconeogenesis pathway, and a low level of PCK1 was highlighted as a potential predictor for a poor prognosis in HCC patients [65].

Notably, high expression levels of miR-20a-3p, miR-636, and miR-7-5p and high expression of *PCK1* were implicated in OXA resistance and poor prognoses of patients with mCRC because the epithelial-to-mesenchymal transition (EMT) pathway is considered an alternative pathway in the development of OXA resistance [66].

Furthermore, we also predicted that the small-molecule compound, mersalyl, would target PCK1 which induces the *VEGF* gene in invasive tumor cases. Our results are also consistent with GeneMania results in PCK1 interactions with the *VEGF* gene, and earlier research revealed a connection between the VEGF inducer gene and the angiogenesis process and PCK1. This line of reasoning showed that mersalyl can also induce PCK1 in the PPAR pathway [67].

The tumor microenvironment (TME) plays an important role in cancer progression of metastatic cancer, and tumor-associated macrophages (TAMs) are important components of the TME. High TAM levels are associated with invasion, migration, and interleukin- (IL-) 6 for tumor progression of CRC metastasis [68]. Tumor infiltration is associated with six cell types: B cells, CD8^+^ cells, CD4^+^ cells, macrophages, neutrophils, and dendritic cells [69]. Our results showed that the *PCK1* gene was consistently associated with CD4^+^ cells and macrophages. This means that the *PCK1* gene can be a tumor prognostic marker for mCRC.

Results of this study showed that PCK1 is associated with overall survival and disease-free survival genes and the prognosis of CRC patients. Some previous studies also showed that miR-20a-3p, miR-636, and miR-7-5p targeted PCK1 in the PPAR pathway and the small-molecule compound, mersalyl, mediated OXA resistance in CRC; results are consistent with those of a previous study.

Nevertheless, some limitations exist in our study. It was difficult to collect sufficient OXA-treated CRC patient samples and conduct in vitro and in vivo studies and to find a suitable public database to evaluate the clinical significance of miR-20a-3p, miR-636, and miR-7-5p targeting PCK1 in terms of expression levels and CRC progression. We believe that miR-20a-3p, miR-636, and miR-7-5p targeting PCK1 in the PPAR pathway and mersalyl play important roles by mediating OXA sensitivity in CRC progression.

## 5. Conclusions

We concluded that miR-7-5p, miR-636, and miR-20a-3p target PCK1 in the PPAR signaling pathway and the small-molecule compound, mersalyl, might be involved in overcoming OXA-resistant CRC. Our findings may provide novel directions and strategies for CRC therapies.

## Figures and Tables

**Table 1:** Upregulated genes with protein-protein interactions.

No.	Gene symbol	Degree
1	*IGF2*	7.0
2	*THBS1*	6.0
3	*VIM*	5.0
4	*IRS1*	4.0
5	*IGFBP1*	4.0
6	*FSTL1*	4.0
7	*SPON1*	4.0
8	*FYN*	4.0
9	*PF4*	4.0
10	*THSD4*	3.0
11	*XAF1*	3.0
12	*ISG20*	3.0
13	*RTP4*	3.0
14	*IFIT2*	3.0
15	*MYLK*	3.0
16	*ALPL*	3.0
17	*TFF2*	2.0
18	*IGFBP2*	2.0
19	*HSPB3*	2.0
20	*PRSS23*	2.0
21	*FMN1*	2.0
22	*CD36*	2.0
23	*TNFRSF14*	2.0
24	*BTLA*	2.0
25	*SSPN*	2.0
26	*GNG11*	2.0
27	*BDKRB2*	2.0
28	*CASK*	2.0
29	*DIO3*	2.0
30	*ALPPL2*	2.0

**Table 2:** Downregulated genes with protein-protein interactions.

No.	Gene symbol	Degree
1	*ABCC2*	5.0
2	*AKR1B1*	5.0
3	*FABP1*	4.0
4	*AKR1C3*	4.0
5	*DHRS9*	4.0
6	*HGD*	4.0
7	*ABCB1*	4.0
8	*HLA-DQB1*	3.0
9	*FUT3*	3.0
10	*TGFB1*	3.0
11	*UNC13A*	3.0
12	*TFF3*	3.0
13	*AKR1C1*	3.0
14	*HBE1*	3.0
15	*NTS*	3.0
16	*VIL1*	3.0
17	*ANG*	3.0
18	*SERPINB2*	2.0
19	*KIFAP3*	2.0
20	*GJA1*	2.0
21	*ST3GAL1*	2.0
22	*HBG1*	2.0
23	*ST6GALNAC1*	2.0
24	*PCK1*	2.0
25	*SYT1*	2.0
26	*CADPS*	2.0
27	*AKR1B10*	2.0
28	*ACE2*	2.0
29	*MVP*	2.0
30	*SLCO2B1*	2.0

**Table 3:** Enriched KEGG pathways.

KEGG pathway	Count	*p* value	Genes
hsa05144: malaria	3	0.022805391	*TGFB1*, *CD36*, *THBS1*
hsa03320: PPAR signaling pathway	3	0.04064955	*FABP1*, *CD36*, *PCK1*
hsa04920: adipocytokine signaling pathway	3	0.044008403	*IRS1*, *CD36*, *PCK1*
hsa01100: metabolic pathways	11	0.054188	*ST6GALNAC1*, *AKR1B10*, *HGD*, *DHRS9*, *AKR1C3*, *AKR1B1*, *ALPL*, *PCK1*, *ST3GAL1*, *ALPPL2*, *FUT3*
hsa00790: folate biosynthesis	2	0.065166	*ALPL*, *ALPPL2*
hsa04931: insulin resistance	3	0.094179	*IRS1*, *CD36*, *PCK1*

PPAR: peroxisome proliferator-activated receptor.

**Table 4:** Small molecules for targeted therapy.

Drug	Gene symbol	Gene name	*p* value	FDR
Mersalyl	*PCK1*	Phosphoenolpyruvate carboxykinase 1	0.005	1.000*e* + 1

FDR: false discovery rate.

## Data Availability

Publicly available datasets were analyzed in this study. The GEO dataset is available at https://www.ncbi.nlm.nih.gov/geo/query/acc.cgi?acc=GSE42387, and for the survival analysis, CRC patient sample is available at https://www.ncbi.nlm.nih.gov/geo/query/acc.cgi?acc=GSE41258.

## Abbreviations

CRC: Colorectal cancer
DEG: Differentially expressed gene
OXA: Oxaliplatin
PPAR: Peroxisome proliferator-activated receptor
TNM: Tumor (T), node (N), and metastasis (M).

## Additional Points

*Highlights*. (i) Malaria, peroxisome proliferator-activated receptor (PPAR) signaling, and the adipocytokine signaling pathway are key pathways in oxaliplatin-resistant colorectal cancer- (CRC-) targeted therapy. (ii) The *CD36* and *PCK1* genes are therapeutic targets for oxaliplatin-resistant CRC treatment. (iii) miR-7-5p, miR-20a-3p, and miR-636 may be upstream targets that can be utilized for treating oxaliplatin-resistant CRC. (iv) High expression levels of miR-7-5p, miR-20a-3p, and miR-636 were associated with poor overall survival of CRC patients. (v) miR-7-5p miR-20a-3p and miR-636 which target PCK1-mediated PPAR signaling are novel therapeutic target pathways for treating oxaliplatin-resistant CRC. (vi) The small-molecule compound, mersalyl, is associated with PCK1 to overcome oxaliplatin-resistant CRC

## Supplementary Materials

Supplementary Materials Supplementary Table 1: Upregulated genes. Supplementary Table 2: downregulated genes. Supplementary Table 3: list of micro (mi)RNA target genes. Supplementary Table 4: list of survival-related microRNAs. Supplementary Figure 1: GEO database analysis of normal vs. CRC tissues using the GSE126093 dataset. Expression levels of (A) miR-7-5p, (B) miR-20a-3p, and (C) miR-636 in colorectal cancer patients. *p* < 0.05 was considered significant. Supplementary Figure 2: ROC curve for normal vs. colorectal cancer (CRC) patients. (A) miR-7-5p, (B) miR-20a-3p, and (C) miR-636. *p* < 0.05 was considered significant. Supplementary Figure 3: correlations between differentially expressed *FABP1*, *CD36*, *IRS1*, and *THBS1* genes and immune cell infiltration in primary colorectal cancer (CRC) patients (A-D) (TIMER).
