# Supplementary material for: Identification of Novel miRNAs, Targeting Genes, Signaling Pathway, and the Small Molecule for Overcoming Oxaliplatin Resistance of Metastatic Colorectal Cancer
Source: Biomed Res Int. 2022 Sep 19;2022:3825760. doi: 10.1155/2022/3825760 (PMC9526582; doi:10.1155/2022/3825760)
Supplement: Supplementary Materials — Supplementary Table 1: Upregulated genes. Supplementary Table 2: downregulated genes. Supplementary Table 3: list of micro (mi)RNA target genes. Supplementary Table 4: list of survival-related microRNAs. Supplementary Figure 1: GEO database analysis of normal vs. CRC tissues using the GSE126093 dataset. Expression levels of (A) miR-7-5p, (B) miR-20a-3p, and (C) miR-636 in colorectal cancer patients. p < 0.05 was considered significant. Supplementary Figure 2: ROC curve for normal vs. colorectal cancer (CRC) patients. (A) miR-7-5p, (B) miR-20a-3p, and (C) miR-636. p < 0.05 was considered significant. Supplementary Figure 3: correlations between differentially expressed FABP1, CD36, IRS1, and THBS1 genes and immune cell infiltration in primary colorectal cancer (CRC) patients (A-D) (TIMER). [file 3825760.f1.docx]

**Supplementary Materials**

**Identification of novel miRNA’s, targeting genes, Signaling Pathway, and the Small Molecule for Overcoming Oxaliplatin Resistance of Metastatic Colorectal Cancer**

**Supplementary Tables**

**Supplementary Table 1. Upregulated genes**

| **Gene symbol** | **Gene name** | **logFC** |
| --- | --- | --- |
| *INHBA* | inhibin, beta A | 5.481982 |
| *FSTL1* | follistatin-like 1 | 2.402579 |
| *PRKCDBP* | protein kinase C, delta binding protein | 3.334504 |
| *GSTT2* | glutathione S-transferase theta 2 | 2.460447 |
| *TCEAL8* | transcription elongation factor A (SII)-like 8 | 3.007634 |
| *GUCY1A3* | guanylate cyclase 1, soluble, alpha 3 | 2.007069 |
| *DIO3* | deiodinase, iodothyronine, type III | 1.751646 |
| *UCHL1* | ubiquitin carboxyl-terminal esterase L1 (ubiquitin thiolesterase) | 1.914698 |
| *FMN1* | formin 1 | 1.966449 |
| *HSPB3* | heat shock 27kDa protein 3 | 2.04361 |
| *ECHDC2* | enoyl CoA hydratase domain containing 2 | 2.118129 |
| *IGF2* | insulin-like growth factor 2 (somatomedin A) | 2.419644 |
| *DIO3OS* | DIO3 opposite strand/antisense RNA (non-protein coding) | 1.786462 |
| *EMB* | embigin | 1.740118 |
| *ACOT4* | acyl-CoA thioesterase 4 | 1.403276 |
| *ZNF226* | zinc finger protein 226 | 1.306932 |
| *FOLR1* | folate receptor 1 (adult) | 1.791259 |
| *CD36* | CD36 molecule (thrombospondin receptor) | 1.664976 |
| *PCP4* | Purkinje cell protein 4 | 4.131577 |
| *NHS* | Nance-Horan syndrome (congenital cataracts and dental anomalies) | 1.373737 |
| *LHFP* | lipoma HMGIC fusion partner | 2.287691 |
| *PID1* | phosphotyrosine interaction domain containing 1 | 1.551861 |
| *C11orf20* | chromosome 11 open reading frame 20 | 2.727257 |
| *PALLD* | palladin, cytoskeletal associated protein | 1.253779 |
| *MEIS3P1* | Meis homeobox 3 pseudogene 1 | 1.940789 |
| *TNAP* | TRAFs and NIK-associated protein | 1.174994 |
| *TGFBI* | transforming growth factor, beta-induced, 68kDa | 1.398626 |
| *TFF1* | trefoil factor 1 | 2.185572 |
| *BTLA* | B and T lymphocyte associated | 1.023593 |
| *SPON1* | spondin 1, extracellular matrix protein | 1.099532 |
| *KCNJ8* | potassium inwardly-rectifying channel, subfamily J, member 8 | 1.09261 |
| *SCARA3* | scavenger receptor class A, member 3 | 1.91774 |
| *LOC728392* | uncharacterized LOC728392 | 1.920335 |
| *FAM189A1* | family with sequence similarity 189, member A1 | 1.24569 |
| *CD200* | CD200 molecule | 1.76698 |
| *LOC440335* | uncharacterized LOC440335 | 2.462685 |
| *PTPRO* | protein tyrosine phosphatase, receptor type, O | 1.200248 |
| *LGR6* | leucine-rich repeat containing G protein-coupled receptor 6 | 1.473204 |
| *CCNYL1* | FIbroblast growth factor 18 | 1.105648 |
| *S100A13* | S100 calcium binding protein A13 | 1.448614 |
| *TNFRSF14* | tumor necrosis factor receptor superfamily, member 14 | 1.210727 |
| *EHD2* | EH-domain containing 2 | 1.271033 |
| *CRIP1* | cysteine-rich protein 1 (intestinal) | 1.291725 |
| *FAM84A* | family with sequence similarity 84, member A | 1.290848 |
| *VIM* | vimentin | 2.017817 |
| *NRXN3* | neurexin 3 | 1.382016 |
| *CASK* | calcium/calmodulin-dependent serine protein kinase (MAGUK family) | 2.747028 |
| *AMIGO2* | adhesion molecule with Ig-like domain 2 | 2.68809 |
| *THBS1* | thrombospondin 1 | 1.388448 |
| *C16orf48* | chromosome 16 open reading frame 48 | 1.833948 |
| *PDE4B* | phosphodiesterase 4B, cAMP-specific | 1.000272 |
| *PRSS23* | protease, serine, 23 | 1.904507 |
| *APOBEC3F* | apolipoprotein B mRNA editing enzyme, catalytic polypeptide-like 3F | 1.408767 |
| *C1orf186* | chromosome 1 open reading frame 186 | 1.410003 |
| *BICC1* | bicaudal C homolog 1 (Drosophila) | 1.520694 |
| *SOD3* | superoxide dismutase 3, extracellular | 1.639266 |
| *IFIT2* | interferon-induced protein with tetratricopeptide repeats 2 | 1.434587 |
| *ZNF226* | zinc finger protein 226 | 1.331977 |
| *ABLIM2* | actin binding LIM protein family, member 2 | 1.246252 |
| *NR2E3* | nuclear receptor subfamily 2, group E, member 3 | 1.217921 |
| *C4orf7* | chromosome 4 open reading frame 7 | 2.303478 |
| *CHADL* | chondroadherin-like | 1.207938 |
| *ZNF185* | zinc finger protein 185 (LIM domain) | 1.344159 |
| *TNNC1* | troponin C type 1 (slow) | 1.448922 |
| *MFAP5* | microfibrillar associated protein 5 | 1.852857 |
| *CDH11* | cadherin 11, type 2, OB-cadherin (osteoblast) | 1.072416 |
| *BTBD16* | BTB (POZ) domain containing 16 | 1.336752 |
| *GNG11* | guanine nucleotide binding protein (G protein), gamma 11 | 2.031765 |
| *ARHGEF17* | Rho guanine nucleotide exchange factor (GEF) 17 | 1.030763 |
| *TFF2* | trefoil factor 2 | 2.267228 |
| *IGFBP1* | insulin-like growth factor binding protein 1 | 2.361942 |
| *ALPP* | alkaline phosphatase, placental | 2.392957 |
| *BDKRB2* | bradykinin receptor B2 | 1.56302 |
| *IRS1* | insulin receptor substrate 1 | 1.043941 |
| *PF4* | platelet factor 4 | 1.05208 |
| *CDK14* | cyclin-dependent kinase 14 | 1.158384 |
| *SLC38A5* | solute carrier family 38, member 5 | 1.40883 |
| *STAG2* | stromal antigen 2 | 1.922278 |
| *ISG20* | interferon stimulated exonuclease gene 20kDa | 1.281837 |
| *LGALS8* | lectin, galactoside-binding, soluble, 8 | 1.29637 |
| *DNAH14* | dynein, axonemal, heavy chain 14 | 1.337385 |
| *ALPPL2* | alkaline phosphatase, placental-like 2 | 2.319486 |
| *FGF18* | fibroblast growth factor 18 | 1.256129 |
| *INHBA* | inhibin, beta A | 2.326972 |
| *CCDC80* | coiled-coil domain containing 80 | 1.113528 |
| *RTP4* | receptor (chemosensory) transporter protein 4 | 1.919823 |
| *DLEC1* | deleted in lung and esophageal cancer 1 | 1.828396 |
| *LGALS2* | lectin, galactoside-binding, soluble, 2 | 2.112067 |
| *MBNL2* | muscleblind-like 2 (Drosophila) | 1.363508 |
| *SSPN* | sarcospan (Kras oncogene-associated gene) | 1.344501 |
| *SYNE1* | spectrin repeat containing, nuclear envelope 1 | 1.615335 |
| *TREX1* | three prime repair exonuclease 1 | 1.284257 |
| *FRAS1* | Fraser syndrome 1 | 1.353194 |
| *PDZK1IP1* | PDZK1 interacting protein 1 | 1.298774 |
| *FOXP2* | forkhead box P2 | 1.517462 |
| *DDX3Y* | DEAD (Asp-Glu-Ala-Asp) box polypeptide 3, Y-linked | 1.02708 |
| *THSD4* | thrombospondin, type I, domain containing 4 | 1.277167 |
| *ANO1* | anoctamin 1, calcium activated chloride channel | 1.062226 |
| *APBB2* | amyloid beta (A4) precursor protein-binding, family B, member 2 | 1.082276 |
| *TMPRSS3* | transmembrane protease, serine 3 | 1.194572 |
| *ATP6V1E2* | ATPase, H+ transporting, lysosomal 31kDa, V1 subunit E2 | 1.002695 |
| *XAF1* | XIAP associated factor 1 | 1.299673 |
| *ARHGAP23* | Rho GTPase activating protein 23 | 2.926581 |
| *TMEM61* | transmembrane protein 61 | 1.090493 |
| *MYLK* | myosin light chain kinase | 1.123808 |
| *GPRIN2* | G protein regulated inducer of neurite outgrowth 2 | 1.618638 |
| *FYN* | FYN oncogene related to SRC, FGR, YES | 1.35267 |
| *TRIM68* | tripartite motif containing 68 | 1.023804 |
| *DEF6* | differentially expressed in FDCP 6 homolog (mouse) | 1.570227 |
| *TMSB15B* | thymosin beta 15B | 1.398282 |
| *ARHGAP29* | Rho GTPase activating protein 29 | 1.260432 |
| *LOC100506130* | uncharacterized LOC100506130 | 1.019286 |
| *IGFBP2* | insulin-like growth factor binding protein 2, 36kDa | 1.650566 |

FC, fold change.

**Supplementary Table 2 Downregulated genes**

| **Gene symbol** | **Gene name** | **logFC** |
| --- | --- | --- |
| *HBG1* | hemoglobin, gamma A | -3.24053 |
| *HBE1* | hemoglobin, epsilon 1 | -3.80228 |
| *POTEB* | POTE ankyrin domain family, member B | -2.60307 |
| *CADPS* | Ca++-dependent secretion activator | -2.90744 |
| *LOC100289255* | uncharacterized LOC100289255 | -3.88686 |
| *DQX1* | DEAQ box RNA-dependent ATPase 1 | -2.21633 |
| *C21orf81* | ankyrin repeat domain 20 family, member A3 pseudogene | -2.17515 |
| *MAGEA2B* | melanoma antigen family A, 2B | -2.07792 |
| *ANKRD20A9P* | ankyrin repeat domain 20 family, member A9, pseudogene | -1.89309 |
| *TUBAL3* | tubulin, alpha-like 3 | -2.76136 |
| *ANXA10* | annexin A10 | -1.96965 |
| *AGMO* | alkylglycerol monooxygenase | -3.26386 |
| *POTEE* | POTE ankyrin domain family, member E | -2.96544 |
| *COL4A5* | collagen, type IV, alpha 5 | -1.92039 |
| *DAZ2* | deleted in azoospermia 2 | -1.33003 |
| *KIFAP3* | kinesin-associated protein 3 | -1.47837 |
| *IFI16* | interferon, gamma-inducible protein 16 | -1.99405 |
| *ASB9* | ankyrin repeat and SOCS box containing 9 | -1.35751 |
| *DHRS2* | dehydrogenase/reductase (SDR family) member 2 | -3.48789 |
| *RBP1* | retinol binding protein 1, cellular | -2.39327 |
| *DAZL* | deleted in azoospermia-like | -1.48874 |
| *POTEG* | POTE ankyrin domain family, member G | -3.16445 |
| *NPSR1* | neuropeptide S receptor 1 | -1.97656 |
| *HLA-DQB1* | major histocompatibility complex, class II, DQ beta 1 | -1.10495 |
| *AKT3* | v-akt murine thymoma viral oncogene homolog 3 (protein kinase B, gamma) | -1.10096 |
| *ST6GALNAC1* | ST6 (alpha-N-acetyl-neuraminyl-2,3-beta-galactosyl-1,3)-N-acetylgalactosaminide alpha-2,6-sialyltransferase 1 | -1.35927 |
| *FABP1* | fatty acid binding protein 1, liver | -4.5236 |
| *PEG10* | paternally expressed 10 | -2.02487 |
| *HHLA2* | HERV-H LTR-associating 2 | -2.97813 |
| *ABCC2* | ATP-binding cassette, sub-family C (CFTR/MRP), member 2 | -2.83666 |
| *SYTL5* | synaptotagmin-like 5 | -2.69869 |
| *DHRS9* | dehydrogenase/reductase (SDR family) member 9 | -1.18308 |
| *HYAL1* | hyaluronoglucosaminidase 1 | -1.22596 |
| *SYT1* | synaptotagmin I | -1.14576 |
| *AKR1C3* | aldo-keto reductase family 1, member C3 (3-alpha hydroxysteroid dehydrogenase, type II) | -1.80381 |
| *SLC39A5* | solute carrier family 39 (metal ion transporter), member 5 | -1.57442 |
| *C19orf51* | chromosome 19 open reading frame 51 | -1.60389 |
| *GJA1* | gap junction protein, alpha 1, 43kDa | -1.11926 |
| *HPDL* | 4-hydroxyphenylpyruvate dioxygenase-like | -1.64092 |
| *POTED* | POTE ankyrin domain family, member D | -1.9718 |
| *NTS* | neurotensin | -3.88473 |
| *FCGBP* | Fc fragment of IgG binding protein | -2.53797 |
| *SRPX* | sushi-repeat containing protein, X-linked | -1.57942 |
| *RNF217* | ring finger protein 217 | -1.0728 |
| *PLA2G4A* | phospholipase A2, group IVA (cytosolic, calcium-dependent) | -1.4164 |
| *LRIG1* | leucine-rich repeats and immunoglobulin-like domains 1 | -1.79942 |
| *TMPRSS4* | transmembrane protease, serine 4 | -1.24441 |
| *COL4A6* | collagen, type IV, alpha 6 | -1.67249 |
| *POTEG* | POTE ankyrin domain family, member G | -1.55507 |
| *FXYD4* | FXYD domain containing ion transport regulator 4 | -1.12524 |
| *TLCD1* | TLC domain containing 1 | -1.12663 |
| *TMPRSS15* | transmembrane protease, serine 15 | -1.27968 |
| *ANG* | angiogenin, ribonuclease, RNase A family, 5 | -1.58875 |
| *OTC* | ornithine carbamoyltransferase | -2.1939 |
| *CD68* | CD68 molecule | -2.37193 |
| *ST3GAL1* | ST3 beta-galactoside alpha-2,3-sialyltransferase 1 | -1.63536 |
| *AKR1B1* | aldo-keto reductase family 1, member B1 (aldose reductase) | -3.61275 |
| *HMGN5* | high mobility group nucleosome binding domain 5 | -1.47273 |
| *PCK1* | phosphoenolpyruvate carboxykinase 1 (soluble) | -1.49445 |
| *ABCB1* | ATP-binding cassette, sub-family B (MDR/TAP), member 1 | -1.90994 |
| *SERPINB2* | serpin peptidase inhibitor, clade B (ovalbumin), member 2 | -1.00154 |
| *SOHLH2* | spermatogenesis and oogenesis specific basic helix-loop-helix 2 | -2.01318 |
| *SLCO2B1* | solute carrier organic anion transporter family, member 2B1 | -1.03747 |
| *LOC440905* | uncharacterized LOC440905 | -1.00453 |
| *AKT3* | v-akt murine thymoma viral oncogene homolog 3 (protein kinase B, gamma) | -1.33611 |
| *TMEM200A* | transmembrane protein 200A | -1.4642 |
| *MVP* | major vault protein | -1.20603 |
| *SPANXD* | SPANX family, member D | -1.08935 |
| *AKR1B10* | aldo-keto reductase family 1, member B10 (aldose reductase) | -3.68456 |
| *TFF3* | trefoil factor 3 (intestinal) | -1.08983 |
| *ACE2* | angiotensin I converting enzyme (peptidyl-dipeptidase A) 2 | -1.15569 |
| *ANKRD30BP2* | ankyrin repeat domain 30B pseudogene 2 | -2.47757 |
| *AKR1C1* | aldo-keto reductase family 1, member C1 (dihydrodiol dehydrogenase 1; 20-alpha (3-alpha)-hydroxysteroid dehydrogenase) | -3.99522 |
| *DUSP6* | dual specificity phosphatase 6 | -1.58466 |
| *BTN1A1* | butyrophilin, subfamily 1, member A1 | -1.06858 |
| *PDZD3* | PDZ domain containing 3 | -1.01747 |
| *CT45A5* | cancer/testis antigen family 45, member A5 | -1.03249 |
| *C3orf32* | chromosome 3 open reading frame 32 | -1.47554 |
| *TMEM200A* | transmembrane protein 200A | -1.4888 |
| *HGD* | homogentisate 1,2-dioxygenase | -1.32188 |
| *DDIT4L* | DNA-damage-inducible transcript 4-like | -1.02314 |
| *FUT3* | fucosyltransferase 3 (galactoside 3(4)-L-fucosyltransferase, Lewis blood group) | -1.1917 |
| *KLK11* | kallikrein-related peptidase 11 | -1.04675 |
| *FTL* | ferritin, light polypeptide | -1.38185 |
| *MYO1A* | myosin IA | -1.73159 |
| *VIL1* | villin 1 | -1.00681 |
| *FAM198B* | family with sequence similarity 198, member B | -1.5528 |
| *NOXO1* | NADPH oxidase organizer 1 | -1.48468 |
| *HOXD13* | homeobox D13 | -1.04152 |
| *C16orf45* | chromosome 16 open reading frame 45 | -1.18198 |
| *KIAA1199* | KIAA1199 | -1.47593 |
| *RNASE1* | ribonuclease, RNase A family, 1 (pancreatic) | -1.40909 |
| *CELA3B* | chymotrypsin-like elastase family, member 3B | -1.23452 |
| *SAMD3* | sterile alpha motif domain containing 3 | -1.44451 |
| *DCBLD2* | discoidin, CUB and LCCL domain containing 2 | -1.40394 |
| *TGFB1* | transforming growth factor, beta 1 | -1.04417 |
| *PROCR* | protein C receptor, endothelial | -1.53249 |
| *UNC13A* | unc-13 homolog A (C. elegans) | -1.01337 |
| *AKR1C1* | aldo-keto reductase family 1, member C1 (dihydrodiol dehydrogenase 1; 20-alpha (3-alpha)-hydroxysteroid dehydrogenase) | -1.21808 |
| *HOXC9* | homeobox C9 | -1.3515 |
| *KIRREL* | kin of IRRE like (Drosophila) | -1.25758 |
| *PTPRR* | protein tyrosine phosphatase, receptor type, R | -1.44706 |
| *LOC84856* | uncharacterized LOC84856 | -1.56373 |
| *TNFRSF25* | tumor necrosis factor receptor superfamily, member 25 | -1.18308 |
| *H19* | H19, imprinted maternally expressed transcript (non-protein coding) | -1.00301 |

FC, fold change.

**Supplementary Table 3 List of micro (mi)RNA target genes**

| **Gene symbol** | **No. of targets** | **List of miRNAs** |
| --- | --- | --- |
| *PCK1* | 47 | hsa-miR-520f-3p, hsa-miR-5692a, hsa-miR-8485, hsa-miR-885-5p, hsa-miR-20a-3p, hsa-miR-372-3p, hsa-miR-12122, hsa-miR-3692-3p, hsa-miR-543, hsa-miR-7849-3p, hsa-miR-3680-3p, hsa-miR-378i, hsa-miR-4496, hsa-miR-422a, hsa-miR-378a-3p, hsa-miR-4307, hsa-miR-139-5p, hsa-miR-7856-5p, hsa-miR-520e-3p, hsa-miR-378b, hsa-miR-9985, hsa-miR-122-5p, hsa-miR-520d-5p, hsa-miR-7-2-3p, hsa-miR-378c, hsa-miR-7-1-3p, hsa-miR-378e, hsa-miR-3140-3p, hsa-miR-520e-5p, hsa-miR-520a-3p, hsa-miR-6505-5p, hsa-miR-6867-5p, hsa-miR-30c-5p, hsa-miR-524-5p, hsa-miR-3135a, hsa-miR-221-3p, hsa-miR-20b-3p, hsa-miR-5681b, hsa-miR-5087, hsa-miR-520d-3p, hsa-miR-636, hsa-miR-6783-5p, hsa-miR-7-5p, hsa-miR-12120, hsa-miR-6796-3p, hsa-miR-574-5p, hsa-miR-302d-3p |

**Supplementary Table 4 List of survival-related microRNAs**

| **Gene name** | **microRNA name** | **Overall survival (log rank *p*-value)** | **Disease-free survival (log rank *p-*value)** |
| --- | --- | --- | --- |
|  |  |  |  |
| ***PCK1*** | hsa-miR-30c-5p | 0.247 | 0.647 |
|  | hsa-miR-7-5p | **0.035** | 0.212 |
|  | hsa-miR-221-3p | 0.895 | 0.133 |
|  | hsa-miR-139-5p | 0.404 | 0.721 |
|  | hsa-miR-20a-3p | **0.013** | 0.322 |
|  | hsa-miR-422a | 0.837 | 0.925 |
|  | hsa-miR-20b-3p | 0.232 | 0.586 |
|  | hsa-miR-636 | **0.050** | 0.739 |

**Bold p- value <0.05 is significant**

**Supplementary Figures**

**Supplementary Figure 1**


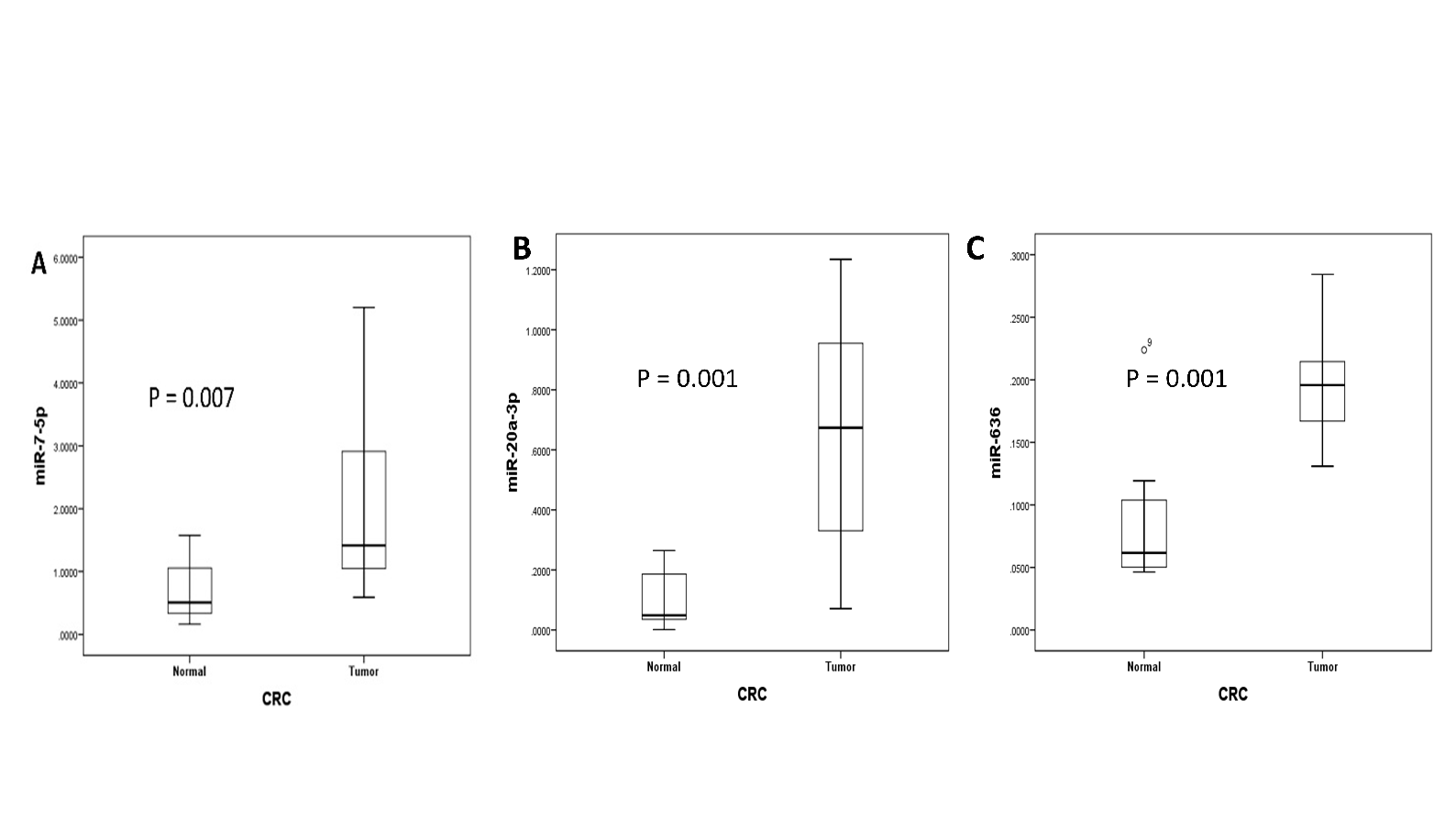


**Supplementary Figure 1 GEO database analysis of normal vs. CRC tissues using the GSE126093 dataset.** Expression levels of (A) miR-7-5p, (B) miR-20a-3p, and (C) miR-636 in colorectal cancer patients**.** *p*<0.05 was considered significant.

**Supplementary Figure 2**


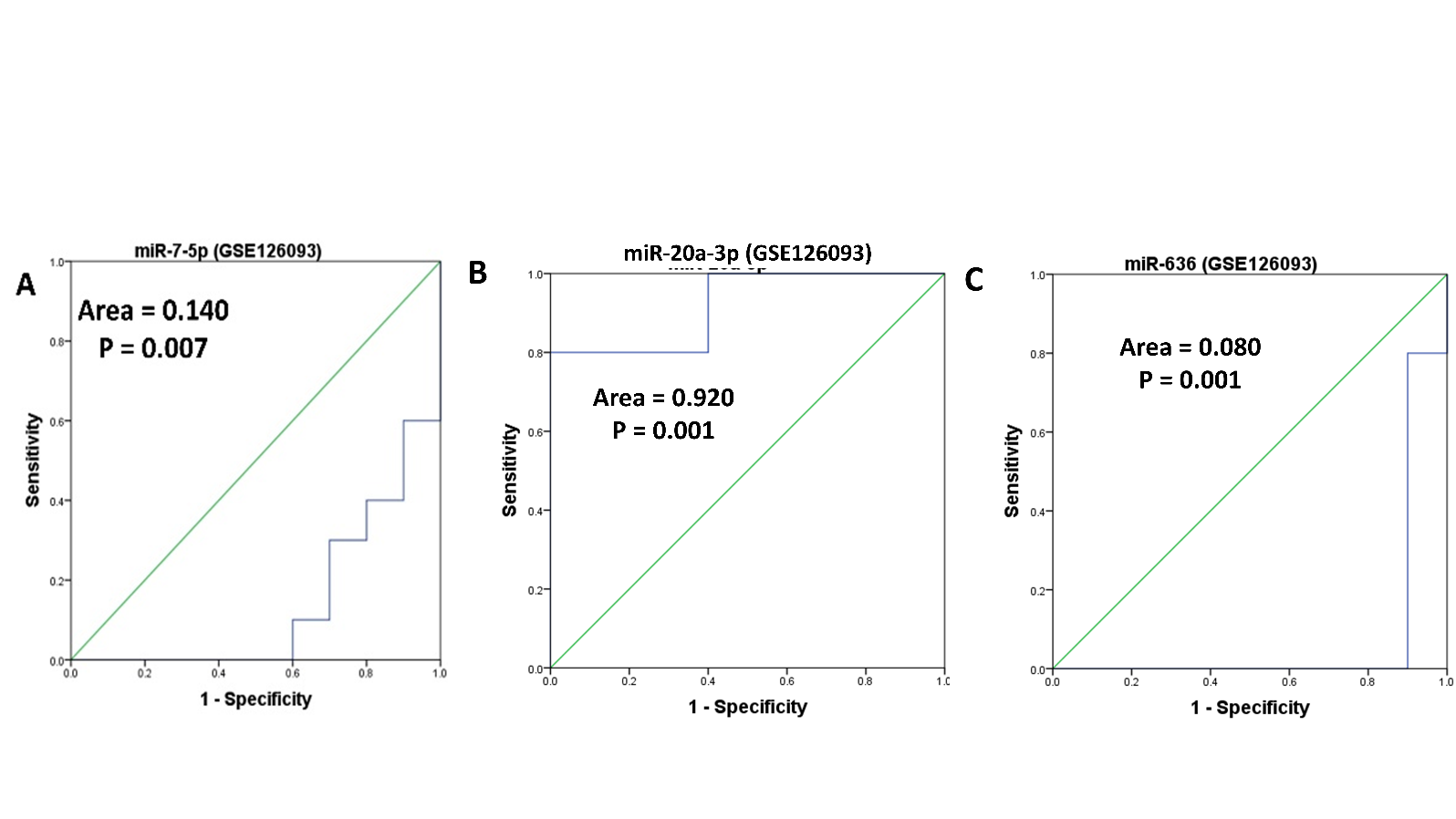


**Supplementary Figure 2. ROC curve for normal vs. colorectal cancer (CRC) patients.** (A) miR-7-5p, (B) miR-20a-3p, and (C) miR-636. *p*<0.05 was considered significant

**Supplementary Figure 3**

**
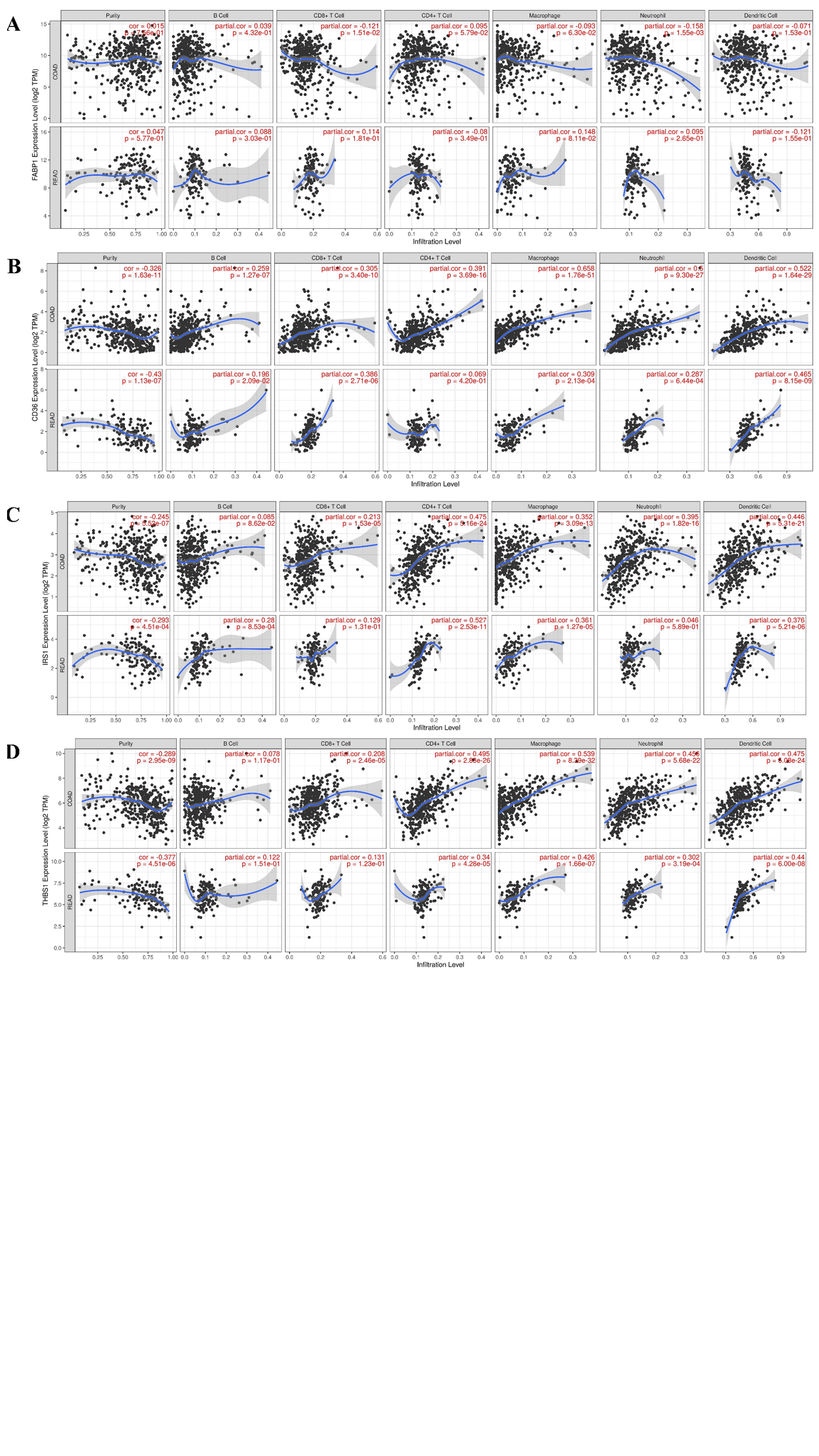
**

**Supplementary Figure 3 Correlations between differentially expressed *FABP1*, *CD36*, *IRS1*, and *THBS1* genes and immune cell infiltration in primary colorectal cancer (CRC) patients (A-D) (TIMER).**
